# Combined Anatomical Variation of Axillary Artery and Biceps Brachii Muscle: A Case Report

**DOI:** 10.15388/Amed.2026.33.1.21

**Published:** 2026-07-22

**Authors:** Dibakar Borthakur, Parul Kaushal, S. B. Ray

**Affiliations:** Department of Anatomy, All India Institute of Medical Sciences, New Delhi, India

**Keywords:** axillary artery, biceps brachii, third head of biceps, pažastinė arterija, dvigalvis raumuo, dvigalvio raumens trečioji galva

## Abstract

Anatomical variations of the axillary artery and its branches are clinically relevant as they may complicate surgeries in the axilla and shoulder. Accessory heads of the biceps brachii can compress key neurovascular structures. The present report describes these variations observed on the left upper limb of a 79-year-old male cadaver during cadaveric dissection. We observed that the usual branches of the 3^rd^ part of the axillary artery as well as the profunda brachii artery originated from the 2^nd^ part of the axillary artery via a common trunk in the left axilla. In addition to that, a small accessory head of the biceps brachii was observed on the left arm originating from the shaft of the humerus. Knowledge about variant branches of the axillary artery is surgically important for establishment of collateral circulation around the scapula following shoulder surgeries. The accessory head of biceps brachii may cause nerve entrapment neuropathy leading to mysterious pain syndrome in the upper limb.

## Abstract (it)

Pažastinės arterijos ir jos šakų anatominiai variantai yra kliniškai reikšmingi, nes jie gali apsunkinti operacijas pažasties ir peties srityje. Dvigalvio žastinio raumens papildomos galvos gali suspausti svarbiausias neurovaskulines struktūras. Šiame tyrime aprašomi šie variantai, pastebėti atliekant 79 metų vyro lavono skrodimą kairėje viršutinėje galūnėje. Norėtume nurodyti, kad įprastos pažastinės arterijos trečiosios dalies šakos, taip pat giliųjų rankos arterijų šakos kyla iš pažastinės arterijos antrosios dalies per bendrą kamieną kairėje pažastyje. Be to, kairėje rankoje buvo nedidelė dvigalvio raumens papildoma galva, kylanti iš žastikaulio stiebo. Žinios apie pažastinės arterijos šakų variantus yra svarbios chirurginiu požiūriu, siekiant sukurti kolateralinę kraujotaką aplink mentę po peties operacijų. Dvigalvio raumens papildoma galva gali sukelti nervo suspaudimo neuropatiją, dėl jos gali kilti paslaptingo skausmo sindromas viršutinėje galūnėje.

## Introduction

The subclavian artery continues as the axillary artery (AA) at the outer border of the first rib, and the AA, in turn, continues as the brachial artery at the lower border of the teres major muscle. The AA is a major artery supplying the upper extremity. The AA is typically divided into three parts with respect to its relation with the pectoralis minor muscle. The 1^st^ part of the AA lies proximal to the pectoralis minor muscle, and it has only one branch, i.e., the superior thoracic artery. The 2^nd^ part of the artery lies deep to the muscle, and its two branches are the thoracoacromial artery and the lateral thoracic artery. The 3^rd^ part of the artery is located distal to the muscle, and its three branches include the anterior and posterior circumflex humeral arteries and the subscapular artery [1]. Anatomical variations of the 3^rd^ part of AA assume special importance as the branches contribute to the anastomosis around the scapula. The branches of the AA also serve as important landmarks during surgeries of the axilla and the shoulder [1,2]. Furthermore, aberrant arterial branches increase the risk of iatrogenic injuries.

The biceps brachii (BB) is a muscle of the anterior compartment of the arm which causes elbow flexion. Its proximal attachment is by means of two heads, hence the name. The long head and the short head attach to the supraglenoid tubercle and the tip of the coracoid process of the scapula, respectively. It also causes supination of the hand by virtue of its distal tendinous attachment on the posterior rough part of the radial tuberosity. Additionally, it plays an important role in forward flexion, abduction and adduction of the arm at the shoulder joint. The intracapsular long head also stabilizes the humeral head in the glenoid fossa and prevents its displacement. The unusual 3^rd^ head of the BB has variable prevalence across different population groups with reported average prevalence of around 22.2% in males and 18.2% in females [3]. The accessory head of BB arises usually from the anteromedial surface of the shaft of the lower 2/3rd of the humerus. On rare occasions, it arises from the anterolateral surface and intertubercular sulcus of the shaft of the humerus, the capsule of the shoulder joint and the coracoid process of the scapula. Generally, the neurovascular supply is identical to the parent muscle [3]. The extra heads of BB once thought to be redundant are now believed to be an important determinant in the pathogenesis of unexplained pain in the arm or shoulder region due to nerve entrapment. We describe here anatomical variation of the AA with a third accessory head of BB on the left arm observed in a male cadaver.

## Methods

The present case was observed in a donated male cadaver aged 79 years during routine prosection undertaken for undergraduate medical teaching. Protocol for dissection was as per Cunningham’s manual of practical Anatomy volume 1, 16^th^ edition. The origin, course and relation of each of the branches of the AA, the brachial plexus and the muscles of the upper extremity were noted. Relevant gross anatomical findings were measured with digital Vernier calipers, and the findings were documented. Ethical clearance was not required as this report was based on the findings on a cadaver donated to the department of Anatomy, and institutional guidelines for the use of human cadavers for medical teaching and research were followed strictly.

## Case report

**Fig. 1A and 1B:**
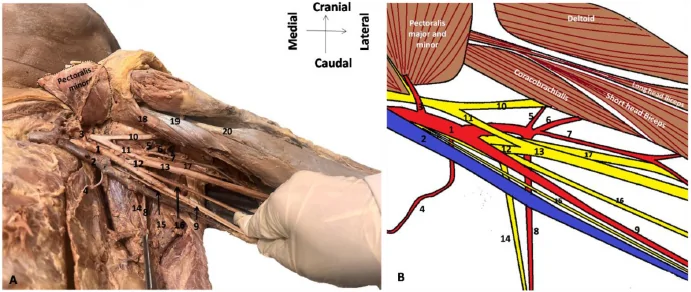
1A. Dissected left axilla showing the major branches of the axillary artery and the brachial plexus. 1B. Schematic drawings of the structures in the left axilla; 1 – axillary artery, 2 – axillary vein, 3 – thoracoacromial artery, 4 – lateral thoracic artery with lateral pectoral nerve, 5 – anterior circumflex humeral artery, 6 – posterior circumflex humeral artery, 7 – variant profunda brachii artery, 8 – variant subscapular artery, 9 – brachial artery, 10 – musculocutaneous nerve, 11 – lateral root of the median nerve, 12 – medial root of the median nerve, 13 – median nerve, 14 – thoracodorsal nerve, 15 – medial cutaneous nerve of the forearm, 16 – ulnar nerve, 17 – radial nerve, 18 – coracobrachialis muscle, 19 – short head of biceps brachii, 20 – long head of biceps brachii.

The 3^rd^ part of the AA on the left side had no branch. Instead, a common arterial trunk arising from the 2^nd^ part provided origin to the branches of the 3^rd^ part of AA. The 1^st^ part of the AA on the left side was 17 mm in length and 11 mm in diameter, and its only branch, i.e., the superior thoracic artery arose 6 mm distal to the outer border of the first rib. The 2^nd^ part of AA measured 19 mm in length and 11 mm in diameter, whose branches were thoracoacromial, lateral thoracic, and a common arterial trunk. The common trunk originated from the posterolateral aspect of the 2^nd^ part of the AA, passed deep to the lateral root of the median nerve for 16.3 mm, and then branched into anterior circumflex humeral, posterior circumflex humeral, subscapular and the profunda brachii arteries. The diameter of anterior circumflex humeral, posterior circumflex humeral, subscapular and the profunda brachii arteries at their origin were 3.5mm, 5.4mm, 6.4mm and 3.7mm, respectively. The posterior circumflex humeral artery accompanied the axillary nerve through the quadrangular space and the profunda brachii artery traversed through the radial groove of the humerus along with the radial nerve (see Fig. 1 A and B). The subscapular artery descended along the lateral border of the scapula and terminated as the circumflex scapular and the thoracodorsal artery. The brachial artery had no branches in the arm, and both the radial and ulnar collateral arteries originated from the variant profunda brachii artery.

**Fig. 2:**
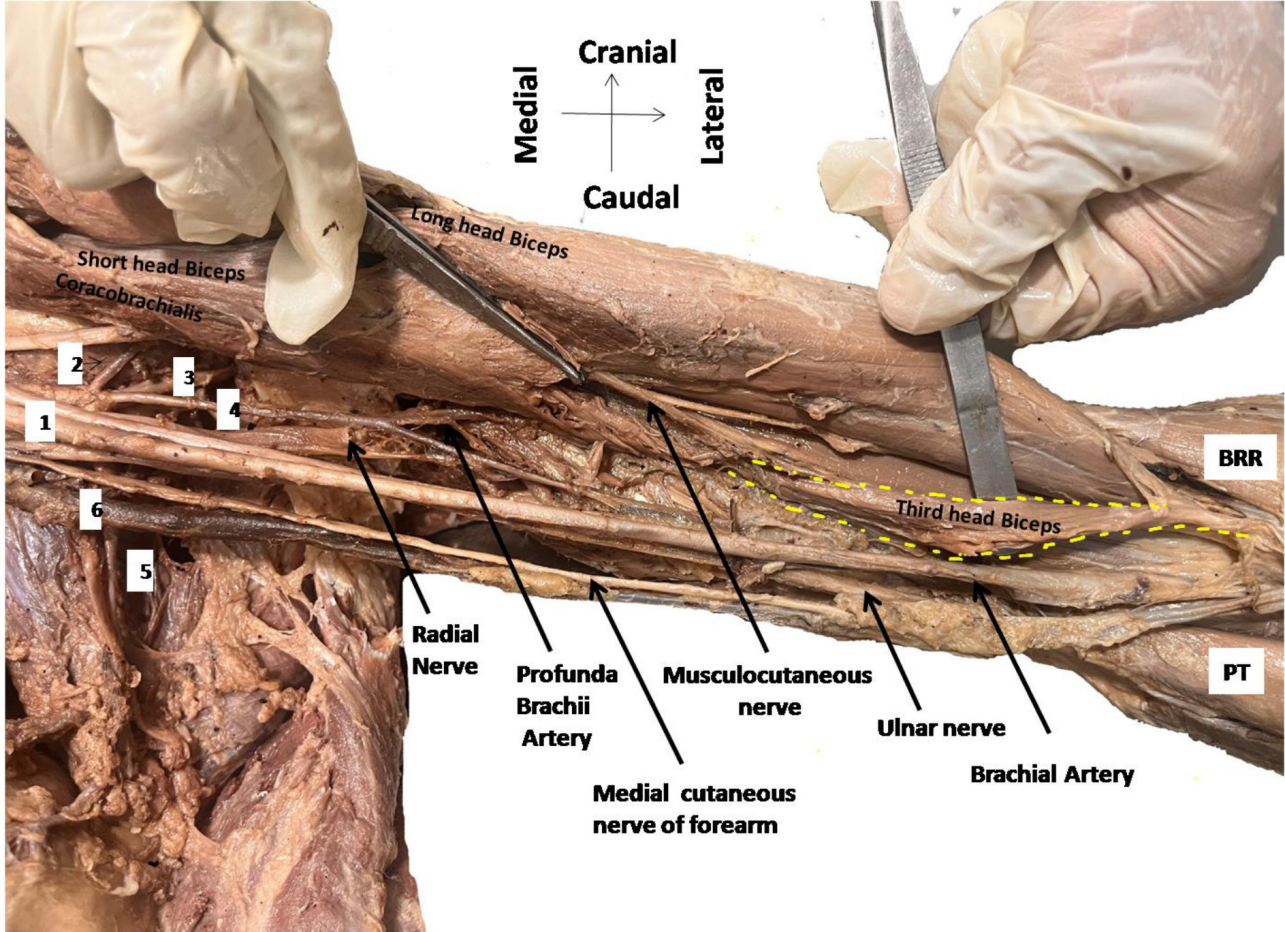
Dissected left axilla and left arm showing the 3^rd^ head of the biceps brachii muscle. 1 – axillary artery, 2 – anterior circumflex humeral artery, 3 – posterior circumflex humeral artery, 4 – profunda brachii artery, 5 – subscapular artery, 6 – axillary vein, BRR – brachioradialis muscle, PT – pronator teres muscle. The 3^rd^ head of the biceps brachii muscle is outlined with a yellow dotted line.

The 3^rd^ accessory head of the BB muscle was observed on the distal third of the left arm which originated from the anteromedial surface of the lower third of the humerus. It moved downward, laterally and blended with the common tendon of BB, and finally inserted on the radial tuberosity. All the three heads of the BB were supplied by the musculocutaneous nerve (see Fig. 2). The right arm revealed neither any aberrant branches of the AA nor any extra heads of the biceps brachii muscle.

## Discussion

One of the reasons for the occurrence of a variant origin of the branches of the 3^rd^ part of AA could be due to faulty angiogenesis. The AA is the continuation of the subclavian artery, and is derived from the lateral branch of the distal part of the seventh cervical intersegmental artery. Branches of the AA are formed from the capillary plexus around the axis artery of the upper limb. Remodelling and maturation of the early undifferentiated capillary networks give rise to the branches of the AA. Incomplete development, abnormal regression of normally retained and abnormal persistence of normally obliterated capillary network around the developing axis artery may result in variant branching pattern [2,4]. Anatomical variation of the 3^rd^ part of the AA in our case resembles some of the patterns of AA variations described in Bergman’s *Comprehensive Encyclopedia of Human Anatomic Variation* [5]. However, the origin of all the three typical branches of the 3^rd^ part of the AA and the profunda brachii artery via a common trunk from the 2^nd^ part of AA has not been described so far. Since the axilla is a common site for varied pathological conditions such as lymph-adenopathy, abscess, aneurysms, trauma and other breast lump in axilla which often require surgical intervention, the presence of an unusual arterial branch may cause complications. This holds particularly true in the present era, when interventional vascular procedures using the transaxillary route are involved [6].

We also observed a 3^rd^ head of the BB on the left arm. During the 5^th^ to 6^th^ week of the intrauterine development, the BB begins to differentiate from the upper limb mesenchyme destined to form the muscles of the anterior compartment of the arm. A muscle develops through various stages of the formation of muscle primordia, some of which undergo apoptosis to eventually give rise to the definite postnatal form of the muscle. During the process of differentiation, few cells of the muscle primordia containing specialized myofilaments may persist without undergoing apoptosis that may later transform into accessory heads [7]. Full range of normal muscle development from an appropriate number of myogenic cells requires proper functioning of a homeobox gene Meox2/Mox2. Abnormally patterned muscle development has been observed in absence of the Meox2 function [8]. Disruption of function of the transcription factor TBX5 that regulates the patterning of muscles in the upper limb is considered to have an additional causal role in upper limb muscle patterning [7].

The contemporary knowledge about accessory heads of BB is quite inconsistent. The reported prevalence of accessory heads of BB is around 9.6%, of which, 8.4% represents the presence of a single accessory head. Up to seven accessory heads of the BB have been reported in the literature [8]. The 3^rd^ accessory head of BB usually arises from the shoulder joint capsule, coracoid process, intertubercular sulcus, anteromedial and anterolateral surfaces of the shaft of the humerus and the brachialis muscle [3,9,10]. The presence of an accessory head of BB may compress key neurovascular structures of the arm such as the musculocutaneous or median nerve. A bulky accessory head may mimic a soft tissue tumor. They can be the reason for mysterious pain syndrome on the arm or shoulder region [3]. Mysterious pain syndrome in the shoulder or the arm region refers to any unexplained pain or undiagnosed pain occurring in these regions whose cause is difficult to ascertain with commonly employed clinical and diagnostic tools. An interesting fact observed about the accessory head of BB is its decreasing prevalence over time which might suggest an evolutionary adaptation [8]. The accessory head of the BB is found to be more prevalent in the male gender, and no association has been observed with the occupation. Incidentally, the 3^rd^ head of BB is frequently found associated with the anatomical variation of the musculocutaneous nerve; however, in our case, the musculocutaneous nerve was normal bilaterally. In addition to that, the presence of a 3^rd^ head of the BB – when present – may abnormally mobilize the fractured segments of the humerus resulting in mal-union [11]. The 3^rd^ head of the BB with a modest incidence in the general population has been implicated in the occurrence of the entrapment neuropathies in the shoulder and arm regions, thereby indicating its role in the mysterious pain syndrome. The 3^rd^ head of the BB is a recognized pain generator in the arm and shoulder region by compressing key neurovascular structures. It is recommended that the presence of a variant accessory muscular head of BB should be considered and evaluated in unresolved cases of mysterious pain in the shoulder or arm region [12,13].

## Conclusion

The coexistence of combined vascular and muscular variation as observed in the present case indicates a faulty development and differentiation of mesoderm of the upper limb. Awareness about variant branches of the axillary artery is imperative for establishing a collateral circulation around the scapula following major surgeries in the shoulder region. The presence of an accessory head of biceps brachii should be considered while dealing with cases of peripheral nerve entrapment or mysterious pain syndrome in the upper limb.
